# Supplementary material for: Genome-Wide Characterization and Expression of the Hsf Gene Family in Salvia miltiorrhiza (Danshen) and the Potential Thermotolerance of SmHsf1 and SmHsf7 in Yeast
Source: Int J Mol Sci. 2023 May 9;24(10):8461. doi: 10.3390/ijms24108461 (PMC10218652; doi:10.3390/ijms24108461)
Supplement: Supplementary file 1 [file ijms-24-08461-s001.zip › Supplementary _Figure S and table S.pdf]

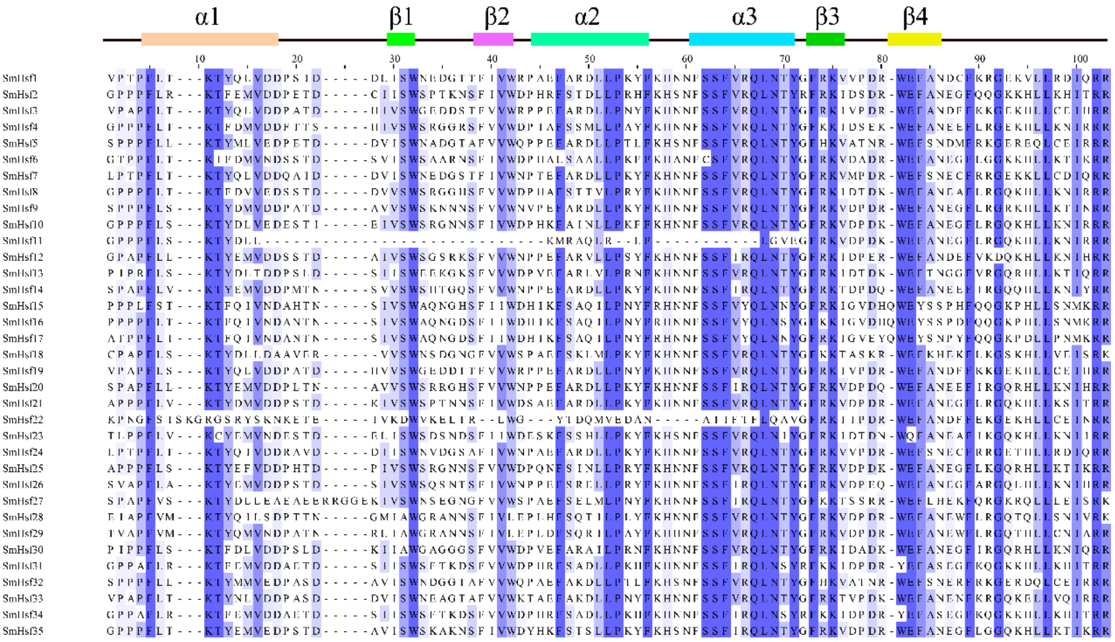

Figure S1 Multiple sequence alignment of DBD domain of SmHsf proteins from *S. miltiorrhiza*. The different colors represent three α-helices and four β-sheets. Sequences were aligned using MEGA with Muscle by default parameters (version 11). Gradient blue colors represent sequence consensus from low to high.

Table S1 Sequence characteristics of heat shock transcription factors (SmHsfs) in *S. miltiorrhiza*.

Gene name	Gene ID	Strand	CDS (bp)	Exons	Protein length (aa)	MW (kDa)	Instability Index	GRAVY	PI	Localization
SmHsf1	EVM0002416	-	873	3	291	31.36	65	-0.61	5.89	Nuclear
SmHsf2	EVM0014513	+	1095	2	365	42.05	76.59	-0.70	5.84	Nuclear
SmHsf3	EVM0027188	-	1026	2	342	37.90	67.8	-0.49	8.22	Nuclear
SmHsf4	EVM0020872	+	1074	4	358	41.64	61.23	-0.91	5.72	Nuclear
SmHsf5	EVM0022616	-	702	2	234	26.51	65.32	-0.80	8.74	Nuclear
SmHsf6	EVM0014116	-	951	**2**	317	34.59	78.99	-0.48	4.88	Nuclear
SmHsf7	EVM0020650	+	1038	3	346	37.89	66.75	-0.59	5.36	Nuclear
SmHsf8	EVM0018201	-	1005	4	335	38.37	65.99	-0.80	5.8	Nuclear
SmHsf9	EVM0013164	-	1527	3	509	56.36	76.38	-0.55	5.26	Nuclear
SmHsf10	EVM0004220	-	987	2	329	37.62	74.27	-0.68	6.29	Nuclear
SmHsf11	EVM0003811	-	780	3	260	29.74	77.53	-0.72	9.19	Cytoplasmic Nuclear
SmHsf12	EVM0013673	+	1449	2	483	53.64	66.78	-0.75	5.36	Nuclear
SmHsf13	EVM0001289	-	1059	2	353	39.75	71.7	-0.62	5.92	Nuclear
SmHsf14	EVM0012238	+	1167	2	389	43.87	68.09	-0.69	5.72	Nuclear
SmHsf15	EVM0000817	-	798	2	266	30.54	71.4	-0.68	6.72	Nuclear
SmHsf16	EVM0024007	+	759	2	253	28.70	74.33	-0.58	5.9	Nuclear
SmHsf17	EVM0014653	+	783	2	261	30.16	78	-0.66	5.77	Nuclear
SmHsf18	EVM0012567	+	564	2	188	21.66	64.12	-0.59	9.76	Mitochondrial Nuclear
SmHsf19	EVM0017861	-	873	2	291	33.42	71.24	-0.63	6.92	Nuclear

Gene name	Gene ID	Strand	CDS (bp)	Exons	Protein length (aa)	MW (kDa)	Instability Index	GRAVY	PI	Localization
SmHsf20	EVM0007953	+	1278	2	426	48.24	68.35	-0.75	5.44	Nuclear
SmHsf21	EVM0002514	+	1509	2	503	55.09	62.01	-0.62	4.78	Nuclear
SmHsf23	EVM0012123	-	1152	2	384	43.55	71.31	-0.65	4.64	Nuclear
SmHsf24	EVM0006191	-	684	2	228	26.43	68.77	-0.73	6.97	Nuclear
SmHsf25	EVM0021531	+	942	2	314	36.13	66.68	-0.79	5.11	Nuclear
SmHsf26	EVM0028007	+	1200	2	400	45.23	68.67	-0.73	5.16	Nuclear
SmHsf27	EVM0004954	+	564	2	188	22.13	65.24	-0.90	9.93	Nuclear
SmHsf28	EVM0003128	-	1011	2	337	37.66	66.76	-0.50	4.89	Nuclear
SmHsf29	EVM0007734	-	1062	2	354	39.55	64.67	-0.72	5.7	Nuclear
SmHsf30	EVM0022005	-	1464	2	488	54.26	66.45	-0.57	5	Cytoplasmic Nuclear
SmHsf31	EVM0010800	+	1026	2	342	39.15	62.02	-0.73	5.39	Nuclear
SmHsf32	EVM0006922	-	675	2	225	25.71	78.75	-0.63	8.32	Nuclear
SmHsf33	EVM0013214	+	810	2	270	29.51	60.26	-0.68	6.34	Nuclear
SmHsf34	EVM0003304	-	882	2	294	33.68	57.61	-0.87	8.43	Nuclear
SmHsf35	EVM0012493	+	1089	3	363	40.94	70.25	- 0.66	4.84	Nuclear

CDS, coding sequece; MW, molecular weight; GRAVY, grand average of hydropathicity; pI, isoelectric point

Table S2 Functional domains analysis of SmHsfs in *S. miltiorrhiza*

Gene name	DBD	HR-A/B	NLS	NES	RD	AHA
SmHsf1	22-115	165-198	(249) LKRLKRSENGNGGGGERMAKDVKSE	N.D.	(242) LFGV	N.D.
SmHsf2	56-149	166-209	(248) GEVLKKRRLA	(199) SHLAAVEKRLRITE	N.D.	N.D.
SmHsf3	21-114	175-202	(305) KKRLHPECNSTAATTNKTRLVLEKDDLG	N.D.	(297) LFGV	N.D.
SmHsf4	40-133	172-206	(312) EDLLKQGFDEGEKLVKDEDAEVYHKNQRF	(271) QMSELEALALQMQ	N.D.	N.D.
SmHsf5	18-111	140-182	(210) EKEKKRKRAHE	N.D.	(200) LFGV	N.D.
SmHsf6	28-121	155-188	(116) KTIKRRRNTS	(274) DPITDDVLEMLLSEED	N.D.	N.D.
SmHsf7	23-116	171-205	(6) VDRSGSGESTAGDAPRSLPTPFLTKTYQL	N.D.	(281) LFGV	N.D.
SmHsf8	34-127	167-202	(220) HKEKRKELEEAISKKRRRPI	(161) KLVLVTELVKLRQQQ	N.D.	N.D.
SmHsf9	14-107	134-171	(212) RHISGANKKRRLPNQDEESLARKLSITS	(167) SQLQTVGQRVHVMEQ	N.D.	(444)NDVFWEQFLPESPLI
SmHsf10	38-131	153-248	(235) IGKKRRKKID	N.D.	N.D.	N.D.
SmHsf11	38-94	107-202	(189) IGKKRRKKID	(226) EAQDFDDLKVGEWD	N.D.	N.D.
SmHsf12	16-109	125-180	(207) YNKKRRLPQ	N.D.	N.D.	(429)DVFWEQFLTERPGC
SmHsf13	46-139	145-181	(23) ESLPLRPPPSVASFDSPRPLASPPIPRFLSKTY	N.D.	N.D.	N.D.
SmHsf14	10-103	120-148	(193) EFQRKKRRSVALNF	N.D.	N.D.	(362) NDVFWQQFLTEAP
SmHsf15	24-118	136-183	(114) NMKRRSLQMSPTPPRQKNSLSP	(245) QHAIAIAQDFQVWLTE	N.D.	N.D.
SmHsf16	22-116	125-162	(111) SNMKRRALQM	N.D.	N.D.	N.D.
SmHsf17	13-107	125-172	(102) PNMKRRSEQNNPTPPRQKNSLSP	(242) QHAIAIAQDFQVWLIE	N.D.	N.D.
SmHsf18	16-108	130-159	(6)RVGSKPHKMKCPAPFLSKTYDLLDAAVERVVS	(152) QISHFKAVEMRLLE	N.D.	N.D.
SmHsf19	21-114	159-189	(258) KKRLHSEFNSSNKSRLVLEKDDLGLNLMP	N.D.	(249) LFGV	N.D.
SmHsf20	10-103	114-171	(193) FPNKKRRLLALQY	N.D.	N.D.	(358)DMFWQQFLTEAPAS
SmHsf21	29-122	150-181	(232) EGNKKRRLKQD	(265) EAAKAMLRQIMKLD	N.D.	(440)SDPFWEKFLQSPPPV
SmHsf22	2-88	146-185	N.D.	N.D.	N.D.	N.D.
SmHsf23	10-103	130-183	N.D.	(177) QQMLSFIVMAMQSPE	N.D.	(304) NDAVLEQLLLPYPST
SmHsf24	22-115	129-160	(172)RARACKLYMTENVKPLELLPMTRFCQEMTR	(210) SAEHHMDLELRLPGD	N.D.	N.D.

Gene name	DBD	HR-A/B	NLS	NES	RD	AHA
SmHsf25	19-112	145-195	(105) LLKTIKRRKQT	N.D.	N.D.	N.D.
SmHsf26	11-104	121-177	(198) HDRKRRFPG	(243) ELLDQIDSSMSLWE	N.D.	(335)NDVFWEQFLTENPGS
SmHsf27	18-116	135-170	(13) RRKMKSPAPFVSKTYDLLEAEAEERRGGEK	(162) QIAHFKALQLSLLQ	N.D.	N.D.
SmHsf28	21-114	137-173	N.D.	N.D.	N.D.	N.D.
SmHsf29	15-108	118-164	(190) RLTPDKKRKLM	N.D.	N.D.	N.D.
SmHsf30	93-186	212-259	N.D.	N.D.	N.D.	N.D.
SmHsf31	63-156	180-229	(256) GMSKKRRRVACE	(207) DMAAVRERLRVTE	(231) LFGV	N.D.
SmHsf32	17-110	134-180	(150) ENKRLKRENDALNSELLLLLKRKCQQL	(200) QALKLFGVRLEVGSE	(204) LFGV	N.D.
SmHsf33	6-99	149-186	(238) KKRGCCSGGGADLCGPHKKQMKPW	(184)DELIAFLTQCVKVAPDE	N.D.	N.D.
SmHsf34	61-154	175-220	(26) RVKEEEESFMFSAEEEEGDQNQPPKAMPME	(201) SSMAAVRERLRVTE	N.D.	N.D.
SmHsf35	39-132	155-205	(125) LLKTIKRRRNVM	N.D.	N.D.	N.D.

DBD, DNA binding domain; HR-A/B, Oligomerization domain; NLS, Nuclear localization signal; NES, Nuclear export signal; AHA, Transcriptional activation domain; RD, Repressor domain; N.D., not detected. For the NLS, NES, RD and AHA column, the numbers in parenthesis are the start site of the functional domain.

Table S3 The detailed information of conserved motifs in SmHsfs by MEME tools

Motif	E-value	Width	Best Possible Match
1	5.7e-1034	41	FVVWDPPEFSRDLLPKYFKHNNFSSFVRQLNTYGFRKVDPD
2	4.2e-513	29	RWEFANEGFLRGQKHLLKNIRRRKTSSSS
3	7.9e-473	29	PPPFLTKTYEMVDDPSTDSIISWSRDGNS
4	5.7e-255	41	CVESGRFGLEEENERLRREKNVLMAELVKLRQQQQSTRAQL
5	9.6e-152	29	ZRLQGTEQKQQQMMSFLAKALQNPDFLQQ
6	3.1e-022	11	PQPMEGLHEAG
7	1.0e-019	15	NDVFWEQFLTEAPGS
8	1.3e-014	23	JVQHKEKKKEIEEAIGKKRRKKI
9	3.0e-016	50	VPHHHHPITGQGFFPYPSRLSISPTDSDDHPWGESPPPLASPTGGGYHSV
10	1.1e-009	41	DHQELVFQDREGGDDHGNYISFGGGVKVEAQDFDDLKVGEW
